# 肺大细胞神经内分泌癌分子亚型和精准治疗的研究进展

**DOI:** 10.3779/j.issn.1009-3419.2025.102.06

**Published:** 2025-03-21

**Authors:** Yuchao FENG, Xiaohong CAO

**Affiliations:** ^1^030001 太原，山西医科大学; ^1^Shanxi Medical University, Taiyuan 030001, China; ^2^032299 汾阳，山西省汾阳医院; ^2^Shanxi Fenyang Hospital, Fenyang 032299, China

**Keywords:** 肺肿瘤, 肺大细胞神经内分泌癌, 分子分型, 靶向治疗, 免疫治疗, 精准治疗, Lung neoplasms, Pulmonary large cell neuroendocrine carcinoma, Molecular subtype, Targeted therapy, Immunotherapy, Precision therapy

## Abstract

肺大细胞神经内分泌癌（large cell neuroendocrine carcinoma, LCNEC）是有独特特征的高级别神经内分泌肿瘤，其治疗多借鉴小细胞肺癌与非小细胞肺癌治疗方案。近年来其发病率呈上升趋势，其预后受个体、临床分期及治疗方式等多因素交互影响，异质性显著。在分子亚型的研究中依据RB1、TP53等关键基因突变划分出多个亚组，基因组亚型与生存率、化疗反应及精准治疗的疗效相关。靶向治疗挖掘出多个靶点，药物疗效不一。免疫治疗进展突出，免疫检查点抑制剂（immune checkpoint inhibitors, ICIs）单用或联合化放疗在不同分期均显成效，却存在过度增殖性疾病风险，精准预后标志物亟待挖掘。本综述针对LCNEC的分子亚型研究和靶向、免疫等精准治疗的最新研究进展进行综述，并指出未来LCNEC治疗将朝着精准化、个体化方向发展。

## Abstract (en)

Pulmonary large cell neuroendocrine carcinoma (LCNEC) is a high-grade neuroendocrine tumor with unique characteristics, and its treatment regimens are primarily derived from those for small cell lung cancer (SCLC) and non-small cell lung cancer (NSCLC). In recent years, the incidence rate has been on the rise, and the prognosis are affected by the interaction of multiple factors such as individual, clinical stage and treatment mode, and the heterogeneity is significant. In the study of molecular subtypes, multiple subgroups were divided according to key gene mutations such as RB1 and TP53, and genomic subtypes were associated with survival, chemotherapy response, and efficacy of precision therapy. Targeted therapy excavates multiple targets, and the efficacy of drugs is different. Immunotherapy has made remarkable progress, and immune checkpoint inhibitors (ICIs) have been effective in all stages of chemotherapy alone or in combination with chemotherapy or radiation therapy, but there is a risk of hyperprogressive diseases, and accurate prognostic markers need to be explored urgently. This review reviews the latest research progress in the study of molecular subtypes and precision therapies such as targeted therapy and immunotherapy of pulmonary LCNEC, and points out that pulmonary LCNEC treatment will develop in the direction of precision and individualization in the future.

肺大细胞神经内分泌癌（large cell neuroendocrine carcinoma, LCNEC）是具有神经内分泌形态、非小细胞癌的细胞学特征和高增殖率的高级别神经内分泌肿瘤^[1]^。近几十年来，LCNEC的发病率一直在增加，而小细胞肺癌（small cell lung cancer, SCLC）和其他类型的非小细胞肺癌（non-small cell lung cancer, NSCLC）的发病率正在下降^[2]^。LCNEC的准确诊断需要综合组织学、细胞学和免疫组化评估，其诊断标准^[1]^包括神经内分泌形态学（小梁、栅栏、类器官嵌套和玫瑰花结形成）；高增殖率（每10个高倍视野>10个有丝分裂）；广泛的区域性坏死；以及至少一种神经内分泌标志物（嗜铬粒蛋白A、突触素和CD56）的免疫组织化学表达。具有显著嗜酸性细胞质的大细胞、大量显著的核仁以及低的核质比这些特征有助于将肺LCNEC与SCLC区分开来。而如果活检样本中包含的组织不足，LCNEC极有可能被错误地诊断为低分化NSCLC、非典型类癌或SCLC。目前LCNEC尚无统一的治疗标准，国内指南推荐内科治疗可采用SCLC化疗方案（2B类推荐证据）^[3]^。考虑到LCNEC的罕见性以及与SCLC和NSCLC的临床病理相似性，LCNEC的治疗方法大多是从SCLC和NSCLC的治疗方法中推断出来的。近年来基因组和转录组学分析表明，LCNEC存在不同的分子亚型，分子亚型研究在优化治疗方案、预测预后、揭示发病机制、设计临床试验和推动个体化精准治疗方面具有重要价值。本文将针对LCNEC的分子亚型研究进展和精准治疗进行综述，并探讨基于分子层面的潜在治疗策略。

## 1 肺LCNEC预后相关因素复杂

### 1.1 个体多维特征影响预后

肺LCNEC患者整体预后不良，尤其是晚期患者，其5年生存率低至8%，诊断时有一半已进展为临床IIIB/IIIC-IV期^[4]^。在基于人群的研究中，年龄、性别、原发肿瘤大小、淋巴结转移和分期被确定为总生存期（overall survival, OS）（3年生存率、5年生存率和总生存率）和肺癌特异性生存期的预后因素^[5,6]^。同样，Khan等^[7]^研究显示，年龄>60岁、男性、肿瘤直径>7 cm、淋巴结和肝转移是与死亡率增加相关的独立危险因素。Eichhorn等^[8]^研究显示淋巴浸润和神经内分泌免疫组化标志物的存在可能是特异性分期系统的预后因素。一项多机构回顾性研究^[9]^调查了251例LCNEC患者行手术切除的预后，结果表明只有淋巴浸润是独立的预后因素。同时表达至少两种神经内分泌标志物与手术切除和辅助化疗后较差的生存率相关^[9,10]^。非三阳性LCNEC患者（即对所有三种神经内分泌标志物均无反应）的5年生存率显著高于三阳性患者^[10]^。以上研究均表明LCNEC的预后受多种因素交互影响，患者个体特征与生存结局紧密关联。

### 1.2 影响预后的其他关键因素

不同的临床分期影响预后，Kinslow等^[11]^提出，I-III期LCNEC的行为与NSCLC相似，而IV期LCNEC更类似于SCLC，提示不同分期患者的预后存在本质差异。对于I、II和III期的LCNEC患者，手术联合化疗是首选治疗方法^[12]^，但IV期患者需以全身治疗为主，预后较早期LCNEC差。此外，治疗方案的差异也影响生存率。Jiang等^[13]^的研究中通过多因素分析确定治疗选择是LCNEC患者独立的生存预后因素。关于化疗方案的选择，Chen等^[14]^进行的一项系统综述发现，在姑息性治疗中，采用SCLC方案较NSCLC方案能带来更高的生存率，提示化疗方案的不同影响预后。Wegner等^[15]^研究也表明在放疗中，立体定向放射外科治疗比全脑放射治疗具有优势。在手术治疗术式的选择上，肺段切除或楔形切除较肺叶切除或双侧肺叶切除显示出更好的预后^[16]^。除此以外，Wan等^[17]^对I期LCNEC患者队列的分析表明肿瘤播散方式也是影响预后的关键因素，气腔播散（spread through air space, STAS）阳性的患者较STAS阴性患者的无病生存期（disease-free survival, DFS）和OS显著缩短，STAS阳性的患者更倾向于从术后辅助治疗中获得潜在获益。Liu等^[18]^研究表明血淋巴细胞与单核细胞比值（lymphocyte-to-monocyte ratio, LMR）是DFS和OS的独立预后预测因子，佐证了免疫炎症指标对预后的影响。

综上，LCNEC患者在肿瘤负荷、身体状况、治疗方式的反应方面表现出显著的异质性。未来研究应聚焦多方面深化探索。一方面，针对个体特征因素，需进一步剖析各因素间的深层交互机制，构建更精准预后评估模型，依据患者个体特征精准预判病情走向；同时深入研究神经内分泌标志物，挖掘其作为靶向治疗靶点的潜力，提升治疗精准度。另一方面，围绕临床分期与治疗方案，应开展大规模多中心临床试验，优化不同分期下手术、化疗、放疗联合方案，如细化I-III期手术化疗具体流程、探索IV期更适配的综合治疗模式；对比不同放疗技术适用场景，明确立体定向放射外科治疗最佳适应证；持续关注新兴治疗技术发展，及时纳入有效手段，为LCNEC患者制定个性化、高效治疗策略，切实改善预后状况。

## 2 LCNEC分子亚型研究：驱动治疗、指导预后与测序革新

### 2.1 RB1和TP53突变是LCNEC亚分类的关键生物标志物

Rekhtman等^[19]^通过高通量测序技术将LCNEC的基因组数据分为三个亚组：一个是TP53和RB1共变异SCLC样亚组；第二组以NSCLC典型突变为特征，如STK11、KRAS突变和KEAP1突变，是非RB1和TP53共变异的NSCLC样亚组；第三个亚组为类癌样LCNEC，该亚组未携带RB1和TP53变异，以低肿瘤突变负荷（tumor mutational burden, TMB）和MEN1变异为特征。Burns等^[20]^通过综合基因组分析进一步定义了Rekhtman等定义的NSCLC样类别为基因组改变，包括SMARCA4、KRAS、FGF3/4/19、STK11、CDKN2A/B、MTAP和CCND1。通过整合基因组和转录组分析，Stewart等^[21]^依据YAP1（Hippo信号通路的关键效应因子）表达将LCNEC分为具有不同分子特征的两种内在亚型。YAP1高表达的LCNEC肿瘤表现出细胞周期和DNA修复通路的激活，其特征为TP53、CDKN2A/B和SMARCA4的共同改变。而YAP1低表达的肿瘤则以神经内分泌分化和代谢过程改变为特征，较常见的表型是TP53和RB1共同突变（类似于SCLC）。以上研究表明RB1和TP53的遗传状况可能是LCNEC分型和治疗选择的重要因素。基因组和转录组学分析揭示了不同的分子亚型，为个体化精准治疗带来了希望。

### 2.2 分子亚型的基因组异质性

George等^[22]^通过全面的基因组学和转录组学分析确定了两个不同的分子亚组：具有双等位基因TP53和STK11/KEAP1改变的I型LCNEC以及TP53和RB1双等位基因失活的II型LCNEC。虽然I型LCNEC与肺腺癌和鳞状细胞癌（NSCLC）具有相同的基因组改变，但却表现出与SCLC相似的神经内分泌特征。而II型LCNEC具有SCLC的突变模式，但神经内分泌标志物的表达较低，与NSCLC相似。George等^[22]^报告中的“type II”对应Rehktoman报告中的“SCLC-type”，“type I”对应“NSCLC-type”，而“type I”和“type II”的神经内分泌标志物和相关基因的表达模式并不同于典型NSCLC和SCLC。类似的，Miyoshi等^[23]^在靶向捕获测序后得出结论，LCNEC的基因组谱与SCLC更相似，包括高频率的TP53和RB1失活突变和PI3K/AKT/mTOR通路的基因变异，但与SCLC相比，LCNEC中RB1突变的发生率显著低于SCLC。以上研究表明分子亚型的研究有助于将SCLC样和NSCLC样LCNEC与典型的SCLC、NSCLC区分开来。

### 2.3 基因组亚型对化疗反应性不同

Zhuo等^[24]^的研究显示在SCLC样LCNEC中，SCLC型化疗方案（依托泊苷+铂类）的疾病控制率（disease control rate, DCR）和总体缓解率（overall response rate, ORR）以及无进展生存期（progression-free survival, PFS）显著高于NSCLC型化疗方案（培美曲塞+铂类）。这可能与其基因组特征（TP53/RB1双等位基因失活）更接近SCLC的分子模式有关，对于NSCLC样LCNEC亚组来说，接受两种化疗方案治疗的患者之间的DCR和ORR没有显著差异，但与NSCLC型方案相比，采用SCLC型化疗方案的患者PFS和OS延长。这可能与化疗对神经内分泌分化程度的调控作用相关，但具体机制需进一步验证。

而Derks等^[25]^的研究结果却截然不同，其表明RB1阳性（RB1野生型）LCNEC患者采用NSCLC型化疗方案（铂类-吉西他滨或-紫杉烷）治疗OS率优于SCLC型化疗方案（铂类-依托泊苷），但RB1阴性（RB1突变）LCNEC患者的预后无差异。目前这两项研究结果的矛盾性可能源于分子分型标准或人群异质性，未来需统一分型标准并扩大样本进一步明确分子分型与化疗疗效的关系。

### 2.4 基因组研究有助于LCNEC患者的精准治疗

基因组分型可帮助识别LCNEC中的分子特征，为精准治疗提供依据。例如，Li等^[26]^研究了19例LCNEC患者的临床病理学、免疫组化和基因组学特征，发现LCNEC中TP53（89.5%）、RB1（42.1%）、APC（31.6%）和MCL1（31.6%）的突变频率较高。进一步研究发现APC突变患者的DFS和OS显著优于无APC突变的患者。APC突变的发现为LCNEC的分子分型提供了新的视角，可能有助于识别预后较好的患者亚群。此外，在Stewart等^[21]^研究表明，YAP1高表达的LCNEC患者对某些靶向治疗（如Hippo通路抑制剂）更敏感，而YAP1低表达的亚型可能对化疗反应更好。这些发现不仅确立了YAP1作为LCNEC亚型分型的生物标志物，并为基于YAP1状态的个性化治疗策略提供了理论依据。同样，Rekhtman等^[19]^对分子亚型的研究表明与NSCLC和SCLC相比，SCLC样和NSCLC样LCNEC亚组的TMB更高，表明LCNEC可能对免疫检查点抑制剂（immune checkpoint inhibitors, ICIs）敏感，为免疫治疗提供理论依据。此外，分子亚型的研究可帮助揭示肿瘤免疫微环境特征（如T细胞浸润、免疫抑制因子表达），为免疫治疗提供新思路。Ferencz等^[27]^研究表明SCLC样LCNEC则可能更多表达VISTA、LAG-3等免疫抑制分子，而免疫抑制分子的高表达可能限制了免疫治疗的疗效。未来针对免疫抑制分子的治疗策略可能会提高SCLC样LCNEC患者免疫治疗的疗效。

综上，基因组学和转录组学研究为LCNEC患者的基因组分型及精准治疗提供了依据。这些研究成果不仅有助于识别预后较好的患者亚群，还为靶向治疗、免疫治疗等提供了重要的理论基础与全新思路，极大地推动了LCNEC治疗领域的发展，为提升患者的治疗效果和预后带来了希望。但在真实世界中，在晚期LCNEC进行组织学诊断后，肿瘤组织通常不足以进行下一代测序（next-generation sequencing, NGS）。未来，游离DNA（cell-free DNA, cfDNA）测序可能是检测体细胞突变和定义LCNEC基因组亚型的可靠方式^[24]^。

## 3 分子靶点与靶向治疗研究进展

LCNEC存在不同的分子亚型，这些亚型在基因和转录组水平上存在差异，独特的基因组改变和转录组图谱为靶向治疗提供了机会，并为LCNEC治疗药物的未来开发框架提供了信息^[28]^。近年来，不断有新的靶向药物被证明有效。例如，Ricco等^[29]^证明，具有BRAF V600E突变的LCNEC患者对BRAF-MEK抑制剂联合治疗有部分反应。Eihuku等^[30]^表明塞普替尼在多发转移的Ret重排LCNEC患者的一线治疗中也展示了良好疗效。下面将针对几种相对常见的靶点进行叙述。

### 3.1 EGFR突变靶向治疗新进展

LCNEC包含潜在的靶向基因改变，包括EGFR突变（±2%）、BRAF突变（±1%）和FGFR1扩增（±3%），这些潜在的治疗靶点在RB1野生型（84%）中比在RB1突变型（50%）肿瘤中更常见^[22]^。吉非替尼和埃克替尼是第一代EGFR-酪氨酸激酶抑制剂（tyrosine kinase inhibitors, TKIs），可有效治疗EGFR外显子19缺失的LCNEC，反应分别持续6^[31]^和8个月^[32]^。同样，在Zhuo等^[24]^研究中携带EGFR L858R突变的RB1野生型患者在化疗后发生疾病进展时接受EGFR-TKIs吉非替尼或厄洛替尼治疗，均达到部分缓解（partial response, PR）。EGFR突变也可以发生在LCNEC-腺癌的混合形式中^[33]^，近年Muto等^[34]^也报道混合型LCNEC患者携带EGFR突变，使用EGFR-TKIs和贝伐珠单抗实现了18个月的PFS。

### 3.2 间变性淋巴瘤激酶（anaplastic lymphoma kinase, ALK）抑制剂在LCNEC治疗中的应用

携带ALK易位的LCNEC患者，并接受选择性TKIs治疗，显示出不同的结果，在Hayashi等^[35]^报道的病例中，患者肺部肿瘤达到部分缓解，而在另一病例中患者却对ALK抑制剂克唑替尼产生耐药^[36]^。布加替尼作为新型ALK抑制剂，在1例LCNEC伴转移性脑肿瘤的患者中也展示良好疗效^[37]^。因此，第二代或第三代ALK-TKIs，例如塞瑞替尼、布加替尼和劳拉替尼，可能是ALK重排和继发性中枢神经系统受累的LCNEC患者的选择。Wiedemann等^[38]^报道1例47岁女性吸烟者诊断为IV期LCNEC，在该患者病程中使用过多种靶向药物，虽然早期应用劳拉替尼，以预防复合突变的发生并改善ALK阳性LCNEC的预后，但是克唑替尼、塞瑞替尼及布格替尼并未取得预期的获益，这也强调了在神经内分泌组织学的高风险环境中需要更有效的抗ALK药物。

### 3.3 PI3K/AKT/mTOR通路靶向探索

在LCNEC肿瘤标本中可见PI3K/AKT/mTOR通路的改变，这可能代表潜在的治疗靶点^[23]^。日本的一项研究^[39]^对SCLC和LCNEC样本进行了基因组分析，报告了LCNEC组中PI3K/AKT/mTOR通路的变化频率与SCLC组相似。在Christopoulos等^[39]^的II期研究中，49例晚期转移的LCNEC患者采用依维莫司（mTOR的TKIs）加紫杉醇和卡铂的一线治疗，展现出一定的有效性且耐受性良好。

### 3.4 DLL3靶点相关药物的疗效探索与局限

DLL3是Notch信号通路的抑制性配体，在正常组织中表达极低，LCNEC患者中DLL3的阳性表达率较高（74%）^[40]^。Saunders等^[41]^证明DLL3靶向抗体-药物偶联物（antibody-drug conjugate, ADC）洛伐妥珠单抗（SC16LD6.5）有效靶向并根除SCLC和LCNEC患者来源的异种移植物肿瘤中表达DLL3的起始细胞，是治疗高级别肺神经内分泌肿瘤的一流ADC。随后，Morgensztern等^[42]^对I期研究应用SC-002（一种DLL-3靶向药物）治疗SCLC和LCNEC，结果显示，SC-002具有全身毒性和有限的疗效，且治疗应答率与DCR均未达到预期水平，提示其在药代动力学、药效学及药物安全性方面存在显著缺陷，难以在现有方案下为患者群体带来足够的临床获益。

展望未来，亟待深入挖掘LCNEC的潜在分子靶点。可借助单细胞测序技术，剖析细胞层面的基因表达差异，锁定更多关键靶点，再运用基因编辑技术验证靶点功能，为新药研发筑牢根基。基于现有靶点药物的疗效差异，应优化靶向药物研发，提高药物有效性、降低毒性，尤其针对ALK、DLL3等靶点药物，克服耐药问题，研发新一代更具优势的制剂。临床研究方面，未来应开展多中心临床试验，基于已知生物标志物，筛选适配患者，精准评估联合治疗效果，进而推动LCNEC靶向治疗迈向精准高效，切实改善患者生存质量与预后。

## 4 LCNEC的免疫治疗研究进展

### 4.1 免疫治疗助力LCNEC预后改善

ICIs主要以靶向程序性细胞死亡受体1（programmed cell death 1, PD-1）及其配体（programmed cell death ligand 1, PD-L1）和细胞毒性T淋巴细胞抗原-4（cytotoxic T lymphocyte-associated antigen-4, CTLA-4）为代表，目前已经改变了晚期LCNEC的治疗策略。

近年来，ICIs作为单一疗法或与化疗联合治疗，在肺癌中取得较大进展。Rekhtman等^[19]^研究表明，与SCLC和NSCLC相比，SCLC样和NSCLC亚组LCNEC的TMB更高，表现出更高的PD-L1表达。Shirasawa等^[43]^研究表明无论PD-L1状态，抗PD-1治疗对晚期LCNEC患者均有效。Sherman等^[44]^的研究纳入了37例晚期LCNEC患者，接受抗PD-1/PD-L1免疫治疗的患者表现出良好的预后，显示LCNEC免疫治疗疗效与NSCLC的免疫治疗疗效相当。有研究^[45,46]^表明ICIs可改善晚期或转移性LCNEC患者的生存结局，在LCNEC中研究免疫治疗策略可能对改善预后至关重要。但均受样本量小和回顾性数据所限，未来应开展更大规模前瞻性研究验证疗效。

### 4.2 ICIs对不同分期LCNEC均成效显著

ICIs治疗对不同临床分期LCNEC患者均有一定疗效。Watanabe等^[47]^报告了1例IA期LCNEC病例，采用帕博利珠单抗进行二线治疗，4年未复发。Kadota等^[48]^报告了1例IIIA期LCNEC病例，采用帕博利珠单抗二线治疗，6个周期后达到完全缓解（complete response, CR），PFS超过4年。同样，Mauclet等^[49]^对1例不可切除的局部晚期LCNEC患者采用姑息性胸部放疗和纳武利尤单抗治疗后肿瘤达到CR。此外，Takimoto等^[50]^报告了1例IVB期LCNEC病例，尽管PD-L1表达为阴性，接受纳武利尤单抗作为三线治疗，疾病仍保持稳定6个月。除此以外，Oda等^[51]^报道了1例混合型LCNEC在多程治疗后接受纳武利尤单抗治疗后获得长期生存的病例，表明在综合治疗后的ICIs治疗对伴有异时性多发转移的LCNEC有效。

### 4.3 免疫联合方案研究进展

免疫治疗联合放化疗仍然是LCNEC的主要研究方向。Song等^[52]^的研究提供了帕博利珠单抗联合化疗治疗晚期LCNEC的抗肿瘤活性的真实临床证据，发现使用帕博利珠单抗联合化疗作为一线治疗与PD-L1表达≥50%的患者PFS改善相关，表明免疫联合化疗可能是有效的一线治疗策略，可改善晚期LCNEC患者的生存结局。同样，Vrontis等^[53]^评估了8例LCNEC患者一线应用依托泊苷加铂类化疗方案联合阿替利珠单抗免疫治疗的疗效，证明该方案可以有效控制肿瘤进展，且该方案带来的缓解具有一定的持久性。Fisch等^[54]^对191例LCNEC患者进行了回顾性研究，结果显示ICIs治疗的中位OS为26.4个月，而其他一线铂类药物双联治疗的患者为9.0个月，非铂类药物化疗为4.0个月。除了一线治疗以外，Agar等^[55]^研究指出纳武利尤单抗是晚期LCNEC患者一线化疗后的有效二线治疗选择。此外，免疫联合新辅助化疗为部分不可切除LCNEC患者争取了手术治疗机会。Chen等^[56]^报告了1例IIIA期LCNEC病例，采用新辅助化疗联合免疫治疗方案，达到PR后进行手术，术后达到主要病理学缓解（major pathological remission, MPR）。Huang等^[57]^报告了1例不可切除IIIB期LCNEC病例，免疫组织化学提示PD-L1表达为10%，采用信迪利单抗+多西他赛+铂类新辅助免疫联合化疗方案治疗后，患者接受了挽救性手术，术后维持上述治疗方案，未见肿瘤复发及转移。除此以外，Zhang等^[58]^的病例中，将帕博利珠单抗联合重组人血管内皮抑制素作为LCNEC患者的一线治疗，进一步讨论了免疫疗法、抗血管生成药物和化疗作为晚期LCNEC一线治疗的可行性。Patel等^[59]^的前瞻性多中心II期临床试验表明抗PD-1联合抗CTLA-4抗体治疗神经内分泌癌患者（包括LCNEC）有一定疗效，提示双免疫联合可能是晚期LCNEC的治疗选择。

### 4.4 免疫治疗成效与潜在风险并存

尽管上述研究表明LCNEC患者对免疫治疗反应良好，但在临床实践中，不同患者对免疫治疗的反应存在显著差异。接受ICIs的患者存在发生过度增殖性疾病（hyperprogressive disease, HPD）的潜在风险。HPD是一种新的进展模式，其特征是肿瘤生长意外加速，Zhang等^[60]^报道了1例LCNEC患者ICIs治疗（阿替利珠单抗）获得长期缓解，但由于接受另一种ICIs药物（斯鲁利单抗），在肿瘤进展后发生HPD。与阿替利珠单抗治疗前相比，斯鲁利单抗治疗前FAT4、SMARCA4、CYLD、CTNNB1和KIT的突变区域发生改变。该病例表明这些突变基因与HPD之间存在潜在关联。具有上述基因的患者在选择ICIs治疗时应谨慎。Han等^[61]^报道了1例LCNEC患者术后化疗后接受贝伐珠单抗、紫杉醇和纳武利尤单抗治疗。尽管PD-L1表达阳性，但患者表现出快速的疾病进展，其潜在原因可能是免疫治疗介入时机过晚，疾病进展强化免疫逃逸机制，过迟启动免疫治疗时，肿瘤细胞已构建稳固的免疫逃逸网络，致使ICIs难以激活免疫系统杀伤肿瘤细胞，最终导致治疗效果不佳、疾病快速恶化。

### 4.5 聚焦免疫治疗预后生物标志物

免疫治疗为LCNEC提供了突破传统治疗局限的新方向，但其疗效预测依赖于多种生物标志物的综合评估，探索可以识别受益人群或反应的疗效和预后标志物，对于实现精准治疗至关重要。以下主要叙述经典和新兴预后标志物。

#### 4.5.1 经典免疫预后生物标志物

PD-L1表达是目前研究较多的免疫检查点相关标志物。在LCNEC相关研究中，Hermans等^[62]^研究表明PD-L1阳性患者的OS优于PD-L1阴性患者。Rösner等^[63]^研究表明，在肺神经内分泌肿瘤中，PD-L1表达水平与免疫治疗反应相关，其可能是LCNEC潜在的预后标志物。但以上均为小型回顾性研究，需要进一步更大规模的临床试验来评估PD-L1状态是否会成为LCNEC免疫治疗潜在的生物标志物。基因组特征方面，TMB作为衡量肿瘤免疫原性的重要指标，高TMB患者因新抗原释放增强免疫应答，生存率和免疫治疗反应率显著提升。Cao等^[64]^研究的结果表明，高TMB与接受免疫治疗的癌症患者更好的生存率相关。Goodman等^[65]^的研究中，TMB高（TMB-H）和TMB低（TMB-L）患者对抗PD-1/抗PD-L1治疗的反应率分别为58%和20%，表明TMB与免疫治疗反应改善之间存在关联。

除此以外，微卫星不稳定性（microsatellite instability, MSI）可能成为潜在的免疫疗效生物标志物。MSI是由于错配修复缺陷（deficient mismatch repair, dMMR）导致DNA复制错误，进而引发微卫星序列长度改变的现象。dMMR导致DNA复制错误无法纠正，进而诱发高TMB，（TMB≥10 mut/Mb）和微卫星高度不稳定性（microsatellite instability-high, MSI-H）。在MSI-H和高TMB的肿瘤中，大量的基因突变会产生众多新抗原，可能增强免疫系统识别和攻击。而PD-1抑制剂通过阻断T细胞抑制信号，恢复其抗肿瘤活性，理论上，在高TMB的肿瘤中，由于存在更多可被免疫系统识别的新抗原，PD-1抑制剂能够更好地发挥作用，使免疫细胞更有效地攻击肿瘤细胞。因此对于预测 PD-1抑制剂等免疫治疗的疗效具有重要的研究价值和临床意义 。例如，Le等^[66]^研究表明，具有dMMR的结直肠癌患者对免疫治疗有反应。dMMR患者的客观缓解率（objective response rate, ORR）为40%，而错配修复正常患者的ORR为0%。类似地，MSI-H的NSCLC患者对ICIs反应率较高，但这一现象在LCNEC中的作用仍需进一步验证。

#### 4.5.2 新兴免疫预后生物标志物

LCNEC的发生发展与免疫及免疫微环境密不可分，免疫微环境具备成为新兴免疫生物标志物的潜力。Christopoulos等^[67]^发现未经治疗的IV期LCNEC患者具有显著的T细胞库改变，这些变化与血淋巴细胞计数呈正相关，表明抗原诱导的T细胞增殖是致病机制。治疗前T细胞库亚群发生显著改变和淋巴细胞计数较高的LCNEC患者表现出更长的生存时间，LCNEC诱导T细胞库的临床相关变化可以在血液检验中检测到，可用于预测预后和治疗目的。Shirasawa等^[43]^的回顾性研究表明，CD8^+^肿瘤浸润淋巴细胞（tumor infiltrating lymphocytes, TILs）的密度是晚期LCNEC患者抗PD-1治疗疗效的预测指标。此外，外周血免疫相关标志物也有重要意义。Shi等^[68]^发现外周血中的中性粒细胞淋巴细胞比值（neutrophil-lymphocyte ratio, NLR）和血小板/淋巴细胞比值（platelet-to-lymphocyte ratio, PLR）是LCNEC患者的独立预后因素。而循环肿瘤细胞（circulating tumor cells, CTCs）和循环肿瘤DNA（circulating tumor DNA, ctDNA）作为液体活检工具，也在识别可能对LCNEC免疫治疗有反应的患者方面发挥作用。CTCs起源于上皮来源的原发性或转移性肿瘤并进入血液，是液体活检的重要组成部分，可以提供转录组学、基因组学和蛋白质组学信息，具有预后预测的替代生物标志物^[69]^。从肿瘤脱落到血液中的DNA通常被称为ctDNA，已被证明可用于预测和监测III/IV期NSCLC患者对ICIs的反应^[70]^。

近年来，免疫治疗为LCNEC带来显著进展。未来，免疫治疗有望优化，应深入探究最佳药物组合与联合模式，依据基因分型、临床分期、PD-L1表达水平等因素精准定策，降低不良反应，针对HPD风险提前识别高危患者并调整用药。单一和通用的预测标志物可能无法准确预测疗效，未来一方面需探索PD-L1+TMB+血清标志物等的组合预测模型，另一方面加速挖掘生物标志物，研究与免疫治疗相关的分子生物标志物并分析获得生存获益患者的分子特征，整合多组学数据构建综合模型，动态监测治疗效果，实现精准高效治疗，改善患者预后。

## 5 未来展望

LCNEC的治疗已从“经验借鉴”迈向“分子驱动”的新阶段。基于基因及转录组学鉴定的精准治疗是未来的发展趋势。未来需要进一步和更大规模的研究来将基因组图谱与对不同治疗策略的反应联系起来，靶向治疗应聚焦于新靶点发现并优化现有药物，免疫治疗方面应聚焦于利用生物标志物筛选免疫获益人群。未来通过多学科协作与技术创新，有望突破现有治疗瓶颈，为患者提供更精准的个体化方案，最终改善生存结局与生活质量。
